# *α*7 Nicotinic Acetylcholine Receptor Stimulation Attenuates Neuroinflammation through JAK2-STAT3 Activation in Murine Models of Intracerebral Hemorrhage

**DOI:** 10.1155/2017/8134653

**Published:** 2017-04-26

**Authors:** Paul R. Krafft, Devin McBride, William B. Rolland, Tim Lekic, Jerry J. Flores, John H. Zhang

**Affiliations:** ^1^Department of Physiology & Pharmacology, Loma Linda University School of Medicine, Loma Linda, CA, USA; ^2^Department of Neurosurgery, Loma Linda University School of Medicine, Loma Linda, CA, USA

## Abstract

Accounting for high mortality and morbidity rates, intracerebral hemorrhage (ICH) remains one of the most detrimental stroke subtypes lacking a specific therapy. Neuroinflammation contributes to ICH-induced brain injury and is associated with unfavorable outcomes. This study aimed to evaluate whether *α*7 nicotinic acetylcholine receptor (*α*7nAChR) stimulation ameliorates neuroinflammation after ICH. Male CD-1 mice and Sprague-Dawley were subjected to intracerebral injection of autologous blood or bacterial collagenase. ICH animals received either *α*7nAChR agonist PHA-543613 alone or combined with *α*7nAChR antagonist methyllycaconitine (MLA) or Janus kinase 2 (JAK2) antagonist AG490. Neurobehavioral deficits were evaluated at 24 hours, 72 hours, and 10 weeks after ICH induction. Perihematomal expressions of JAK2, signal transducer and activator of transcription 3 (STAT3), tumor necrosis factor-*α* (TNF-*α*), and myeloperoxidase (MPO) were quantified via Western blot. Histologic volumetric analysis of brain tissues was conducted after 10 weeks following ICH induction. PHA-543613 improved short-term neurobehavioral (sensorimotor) deficits and increased activated perihematomal JAK2 and STAT3 expressions while decreasing TNF-*α* and MPO expressions after ICH. MLA reversed these treatment effects. PHA-543613 also improved long-term neurobehavioral (sensorimotor, learning, and memory) deficits and ameliorated brain atrophy after ICH. These treatment effects were reduced by AG490. *α*7nAChR stimulation reduced neuroinflammation via activation of the JAK2-STAT3 pathway, thereby ameliorating the short- and long-term sequelae after ICH.

## 1. Introduction 

Accounting for high mortality and morbidity rates, spontaneous intracerebral hemorrhage (ICH) remains one of the most detrimental stroke subtypes; yet no specific therapy has been proven clinically effective [1, 2]. Poor outcomes following ICH are associated with large hematoma volumes, infratentorial origin, and intraventricular extension of the bleed as well as with advanced age and compromised neurological function of patients upon presentation [3]. Since these factors are mostly inalterable, current investigations aim to manipulate cellular and molecular mechanisms of hemorrhage-induced brain injury, pursuing to ameliorate the clinical sequelae following ICH.

Intraparenchymal hematoma formation leads to mass effect and disruption of brain tissue (primary injury); however, it also triggers subsequent deleterious mechanisms including inflammatory pathways (secondary injury). Neuroinflammation after ICH involves microglia activation, production of inflammatory cytokines, and influx of peripheral immune cells, all contributing to the pathophysiology of secondary brain injury [4, 5]. While microglia are implicated in hematoma clearance, classically activated M1 microglial cells release proinflammatory cytokines, such as tumor necrosis factor-*α* (TNF-*α*) and interleukin-6 (IL-6), thereby attracting peripheral leukocytes to the already compromised brain tissue. Infiltrating leukocytes disrupt the blood-brain barrier (BBB) and aggravate neuroinflammation. Hypothetically, inhibiting excessive neuroinflammation may decrease the extent of brain injury and result in improved neurological function after ICH [6].

Nicotinic acetylcholine receptors are pentameric, ligand-gated ion channels expressed in the central and peripheral nervous systems as well as at the neuromuscular junction, implicated in acetylcholine signaling [7, 8]. The *α*7 nicotinic acetylcholine receptor (*α*7nAChR), specifically, is a homopentameric ion channel, highly permeable to Ca^2+^, which is involved in cellular processes such as cell plasticity and survival as well as in higher brain functions such as learning and memory, anxiety, and locomotion [8]. Additionally, *α*7nAChRs are expressed by macrophages and microglia [7, 9]. Acetylcholine-induced *α*7nAChR stimulation inhibited the release of proinflammatory cytokines by peripheral macrophages in wild-type mice, which was not observed in *α*7 deficient mice [7]. Although it is not entirely understood, *α*7nAChR stimulation has been associated with recruitment and activation of Janus kinase 2 (JAK2) and signal transducer and activator of transcription 3 (STAT3), which negatively regulates TNF-*α* synthesis [10].

Administration of specific *α*7nAChR agonists results in neuroprotection following experimental ICH [11, 12]; however, possible underlying anti-inflammatory mechanisms of this treatment strategy have yet to be explored. For this reason, we aimed to evaluate whether PHA-543613, a specific *α*7nAChR agonist, can attenuate neuroinflammation after ICH via JAK2/STAT3 signaling. We hypothesize that PHA-543613 treatment will decrease hemorrhage-induced neuroinflammation by activation of JAK2/STAT3, thus achieving lasting neuroprotection following experimental ICH.

## 2. Materials and Methods

### 2.1. Animal Surgery and Experimental Groups

The Institutional Animal Care and Use Committee at Loma Linda University approved all experiments. This study was carried out in compliance with the NIH guide for the care and use of laboratory animals. Male CD-1 mice (weight: 30–40 g; Charles River Laboratory, Wilmington, MA) and male Sprague-Dawley rats (weight: 280–350 g; Harlan, Livermore, CA) were housed in a temperature-controlled environment with a 12-hour light/dark cycle and were given free access to food and water. The autologous blood injection model was used to create intracerebral bleeds in mice [13]; however, rats were subjected to intracerebral collagenase injections as previously described [14]. Briefly, mice and rats were anesthetized with a mixture of ketamine (100 mg/kg) and xylazine (10 mg/kg). The rodents were placed prone and secured onto a stereotactic head frame (Kopf Instruments, Tujunga, CA). A 1 mm burr hole was made at established coordinates (mice: 0.2 mm anterior and 2.0 mm right lateral from bregma; rats: 0.2 mm anterior and 2.9 mm right lateral from bregma).

The blood injection model required puncture of the rodent's ventral tail artery and collection of 30 *μ*L blood using a capillary tube. The blood was then transferred to a glass syringe with a 26-Gauge needle, which was inserted through the burr hole and advanced 3.0 mm below the dura. At this position, a microinjection pump (Harvard Apparatus, Holliston, MA) was used to deliver 5 *μ*L of blood at a rate of 2 *μ*L/min. The needle was then lowered to a depth of 3.7 mm, and after a waiting period of 5 minutes, 25 *μ*L of blood was injected into the right striatum.

For the collagenase model, 3.0 U of bacterial collagenase (Type VII-S, Sigma-Aldrich, St. Louis, MO), dissolved in 0.5 *μ*L phosphate buffered saline (PBS), was filled into a glass syringe. The 26-Gauge needle attached to the syringe was then lowered through the burr hole to a depth of 5.6 mm below the dura. Collagenase was delivered into the right striatum at a rate of 0.25 *μ*L/min.

After completed injection of autologous blood or collagenase, the needle was left in place for 10 minutes to minimize backflow along the needle tract. Sham surgery consisted of needle insertion without injection of blood or collagenase.

Thirty-six CD-1 mice and 30 Sprague-Dawley rats were utilized in this study. Twenty-seven mice were subjected to intrastriatal injection of autologous blood. These animals received either intraperitoneal injections of normal saline 1 hour after surgery (Vehicle, *n* = 9), injections of *α*7nAChR agonist PHA-543613 (PHA; Sigma-Aldrich (St. Louis, MO); 12 mg/kg; 0.2 mL; intraperitoneally (Krafft et al., 2012)) 1 hour after surgery (PHA-12 mg, *n* = 9), or injections of *α*7nAChR antagonist methyllycaconitine (MLA; Sigma-Aldrich (St. Louis, MO); 6 mg/kg; 0.2 mL; intraperitoneally (Krafft et al., 2012)) 45 minutes after surgery followed by PHA-543613 administration 15 minutes thereafter (PHA + MLA; *n* = 9). Nine animals were subjected to Sham surgery (Sham). All mice underwent neurobehavioral testing 24 hours after surgery followed by brain tissue collection for Western blotting (*n* = 6 per group) and immunohistochemistry (*n* = 3 per group).

Eighteen rats were subjected to intrastriatal injection of collagenase. These animals received either normal saline administrations (Vehicle; *n* = 6) or injections of PHA-543613 (12 mg/kg; intraperitoneally) on days 1, 2, and 3 after ICH (PHA-12 mg; *n* = 6). Another group received the same treatment regimen of PHA-543613 combined with JAK2 antagonist tyrphostin AG490 (AG490; Sigma-Aldrich (St. Louis, MO); dissolved in DMSO; 5 mg/kg; intraperitoneally [12, 15]), which was administered 15 minutes prior to the PHA treatments (PHA + AG490; *n* = 6). Twelve rats were subjected to Sham surgery, 6 of which received injections of AG490 on days 1, 2, and 3 after surgery (Sham + AG490). All rats underwent neurobehavioral testing at 24 and 72 hours, as well as at 10 weeks, after surgery. Brain tissue was collected at the end of the 10th week for volumetric measurements.

### 2.2. Assessment of Neurobehavioral Deficits

All behavioral tests were conducted in a blinded fashion. Acute deficits in rodents, at 24 and 72 hours after ICH, were evaluated using the modified Garcia neuroscore [12, 16] and the forelimb placement test [17]. The Garcia neuroscore consists of 7 individual tests, evaluating spontaneous activity, axial sensation, vibrissae proprioception, symmetry of limb movement, lateral turning, forelimb walking, and climbing. Each subtest is scored from 0 to 3, with a composite maximum score of 21 (no neurological deficits). The forelimb placement test was used to assess the animal's responsiveness to vibrissae stimulation, and results were expressed as percentage of the number of successful left forepaw placements out of 10 stimulations, normalized to the mean of Sham performance. CD-1 mice subjected to autologous blood injection underwent neurobehavioral testing at 24 hours after ICH induction; Sprague-Dawley rats subjected to collagenase injection were examined at 24 and 72 hours after ICH induction.

Long-term neurobehavioral assessments were conducted within the 10th week after intracerebral collagenase injection in rats. Learning and memory abilities were evaluated using the Morris water maze as previously described [14]. This test required the finding of a slightly submerged platform within a water basin. Following learning trials the platform was removed and an overhead infrared camera, linked to a computer tracking system (Noldus Ethovision, WA, USA), recorded the swim path of each animal, measuring the latency of first platform crossing, frequency of platform crossings, and frequency of correct quadrant crossings. Motor function and proprioception were assessed via the rotarod test. Animals were placed on horizontal cylinders (7 cm diameter, 9.5 cm width; Columbus Instruments, OH, USA) either rotating at a constant velocity or accelerating 2 RPM every 5 seconds (starting at 5 or 10 RPM). The falling latency was recorded. Proprioception was also evaluated via the foot fault test in which animals were allowed to move along a horizontal wire-grid (20 cm × 100 cm) for 2 minutes. The number of left-sided missteps was recorded. Activity and anxiety-like behavior were evaluated using the open field test. Rodents were placed in open-topped opaque boxes (49 × 36 × 45 cm) and an overhead camera, linked to a computer tracking system (Noldus Ethovision, WA, USA), recorded the total distance moved as well as the frequency of corner zone crossings during 30-minute trials (divided into 3-minute intervals).

### 2.3. Western Blot

Western blot was performed as previously described [12]. At 24 hours after surgery, immediately following neurobehavioral testing, anesthetized animals were transcardially perfused with PBS and brains were removed and separated into both hemispheres. The ipsilateral brain hemispheres were homogenized in RIPA Lysis and Extraction Buffer (Santa Cruz Biotechnology, Inc., TX, USA) and centrifuged for 30 minutes at 14,000 g. The protein concentration of the supernatant was measured using a detergent compatible assay (DC protein assay, Bio-Rad Laboratories, CA, USA). Equal amounts of protein (50 *μ*g) were loaded to a 10% SDS-PAGE gel. After gel electrophoresis, transfer to a nitrocellulose membrane, and blocking with 5% nonfat blocking grade milk (Bio-Rad Laboratories, Irvine, CA, USA), the membrane was incubated with the primary antibody overnight followed by the appropriate secondary antibodies (1 : 1000). The primary antibodies were anti-p-JAK2, anti-pSTAT3, anti-TNF-*α*, anti-myeloperoxidase (anti-MPO), and anti-*β*-actin (Santa Cruz Biotechnology, Inc., TX, USA). Proteins on immunoblots were visualized with an ECL Plus chemiluminescence reagent kit (Amersham Bioscience, IL, USA). The blot bands were quantified using ImageJ (NIH) and the results were expressed as ratio of the target band intensity to the band intensity of *β*-actin and then normalized to the mean Sham group ratio.

### 2.4. Immunohistochemistry

At 24 hours after surgery, anesthetized animals were transcardially perfused with PBS followed by 4% paraformaldehyde. The brains were removed, fixed with formalin (for 3 days), and dehydrated with 30% sucrose solution (for 3 days). Brain specimens were then flash frozen and cut into 10 *μ*m slices using a cryostat (CM3050S, Leica Biosystems, Inc., IL, USA). All brain slices between 1 mm anterior and posterior from the center of the hemorrhage were washed with PBS and then incubated with blocking solution (10% normal goat serum in 0.1 M PBS). The slides were incubated with the following primary antibodies: anti-*α*7nAChR and anti-Iba1 (Abcam, MA, USA), as well as anti-MPO (Santa Cruz Biotechnology, TX, USA), and DAPI (Vector Laboratories, Inc., CA, USA). After washing the slides in PBS, the appropriate secondary antibodies (Santa Cruz Biotechnology, TX, USA) were applied for 2 hours at room temperature. Stained slices were evaluated using an OLYMPUS BX51 microscope.

### 2.5. Histology and Volumetric Evaluation

After the 10th week, deeply anesthetized rats were transcardially perfused with PBS followed by 4% paraformaldehyde. The brains were removed and 10 *μ*m tissue slices were obtained as mentioned above and stained with cresyl violet [18]. Ventricular volume, cortical thickness, and volume of basal ganglia were measured using ImageJ. The borders of the mentioned brain structures were delineated using optical dissector principles [19, 20]. The volumes of these structures were then calculated as average size of the delineated areas from sections taken at 1.8 mm, 0.74 mm, 0.14 mm, and −1.06 mm (relative to bregma) and multiplied by the depth between succeeding slices. Cell density loss was evaluated by counting neuronal cells (Nissl stain) in 5 areas (250 *μ*m × 250 *μ*m) per brain hemisphere on different brain sections (1.8 mm, 0.74 mm, 0.14 mm, and −1.06 mm relative to bregma). The relative cell density loss was calculated by subtracting the average cell counts of the ipsilateral hemisphere from the average cell counts of the contralateral hemisphere.

### 2.6. Statistical Analysis

Data is presented as the mean ± standard error of the mean (SEM). Western blot data were analyzed using one-way ANOVA with Tukey post hoc tests. Behavior data were analyzed using one-way ANOVA on ranks with Tukey post hoc tests or repeated measures ANOVA when appropriate. All histological data were analyzed using one-way ANOVA with Student-Newman's post hoc tests. A *p* value less than 0.05 was considered statistically significant.

## 3. Results

### 3.1. PHA-543613 Attenuates Neurobehavioral Deficits and Neuroinflammation at 24 Hours after ICH Induction via Activation of the JAK2/STAT3 Pathway

**Figure 1:**
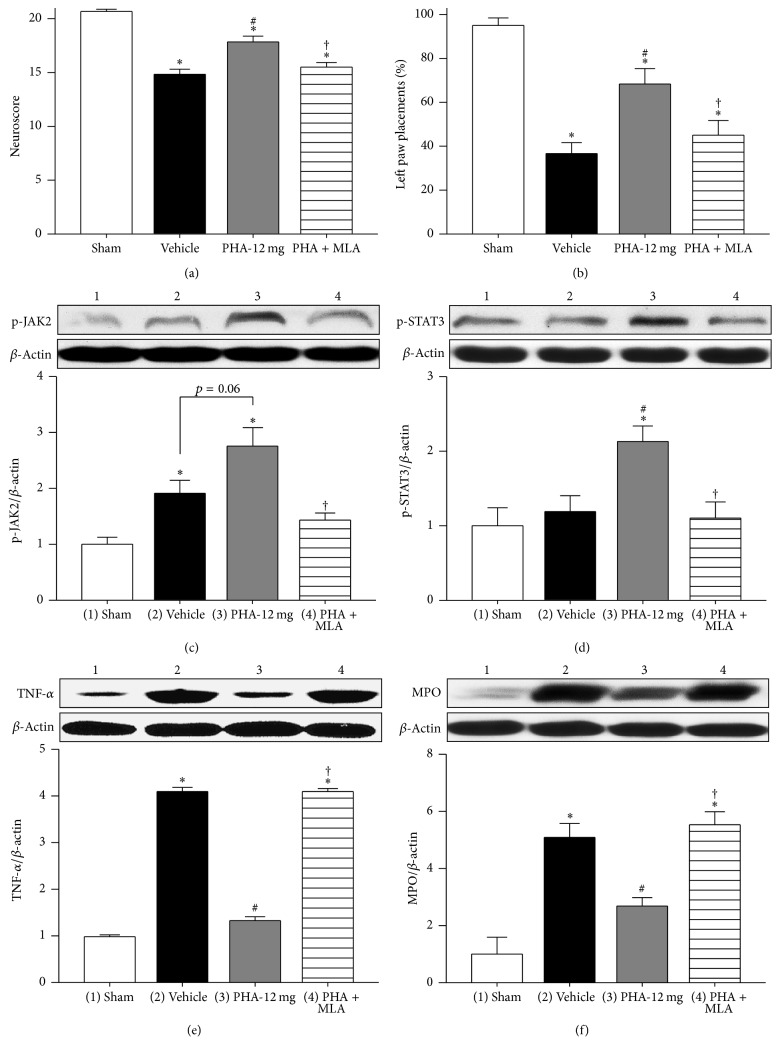
(a) Modified Garcia neuroscore 24 hours after autologous blood injection. *n* = 9/group. (b) Forelimb placing test 24 hours after autologous blood injection. *n* = 9/group. Western blot analysis of p-JAK2/*β*-actin (c), p-STAT3/*β*-actin (d), TNF-*α*/*β*-actin (e), and MPO/*β*-actin (f) 24 hours after autologous blood injection. *n* = 6/group for Western blots. Values are expressed as mean ± SEM. ^*∗*^*p* < 0.05 versus Sham, ^#^*p* < 0.05 versus Vehicle, and ^†^*p* < 0.05 versus PHA-12 mg.

CD-1 mice were subjected to intrastriatal injection of autologous blood and neurobehavioral deficits as well as neuroinflammation were evaluated 24 hours after ICH induction. Animals in the Vehicle group exhibited significantly poorer performances evaluated via the neuroscore (Figure 1(a); *n* = 9/group) and the forelimb placement test (Figure 1(b), *n* = 9/group) when compared to Sham (*p* < 0.05). These neurobehavioral deficits were partially reversed with administration of *α*7nAChR agonist PHA-543613. Animals in the treatment group (PHA-12 mg) performed significantly better in both tests when compared to Vehicle (*p* < 0.05); however, PHA-543613 treated ICH animals did not reach the superior performance scores of Sham animals (*p* < 0.05). Inhibition of *α*7nAChR by MLA reversed the treatment effects. ICH animals in the PHA + MLA group demonstrated significantly poorer performances than those in the PHA-12 mg group (*p* < 0.05); no significant difference was found between the PHA + MLA group and Vehicle (*p* > 0.05).

Molecular changes following ICH induction and administration of *α*7nAChR modulators were evaluated via Western blot (Figures 1(c)–1(f), *n* = 6/group). Intrastriatal injection of autologous blood resulted in significantly increased expression of activated, phosphorylated JAK2 (p-JAK2) within the ipsilateral brain hemisphere (*p* < 0.05 compared to Sham, Figure 1(c)). The expression of p-JAK2 was further increased with PHA-543613 treatment; however, no statistical significance was achieved (*p* = 0.06 compared to Vehicle). MLA administration reversed the treatment effect (*p* < 0.05 compared to PHA-12 mg). The expression of activated, phosphorylated STAT3 (p-STAT3) was similar in the Sham and Vehicle groups (*p* > 0.05, Figure 1(d)). PHA-543613 treatment resulted in a significant increase in p-STAT3 when compared to Sham and Vehicle (*p* < 0.05); however, MLA administration reversed this treatment effect (*p* < 0.05 compared to PHA-12 mg). All CD-1 mice subjected to autologous blood injection demonstrated significantly higher expressions of TNF-*α* when compared to Sham animals (*p* < 0.05, Figure 1(e)). PHA-543613 significantly reduced the expression of TNF-*α* within the ipsilateral brain hemisphere (*p* < 0.05, compared to Vehicle). This treatment effect was reversed by administration of MLA (*p* < 0.05 compared to PHA-12 mg). Similarly, PHA-543613 treatment significantly reduced the expression of neutrophil MPO after ICH induction (*p* < 0.05 compared to Vehicle, Figure 1(f)), which also was reversed by MLA (*p* < 0.05 compared to PHA-12 mg).

**Figure 2:**
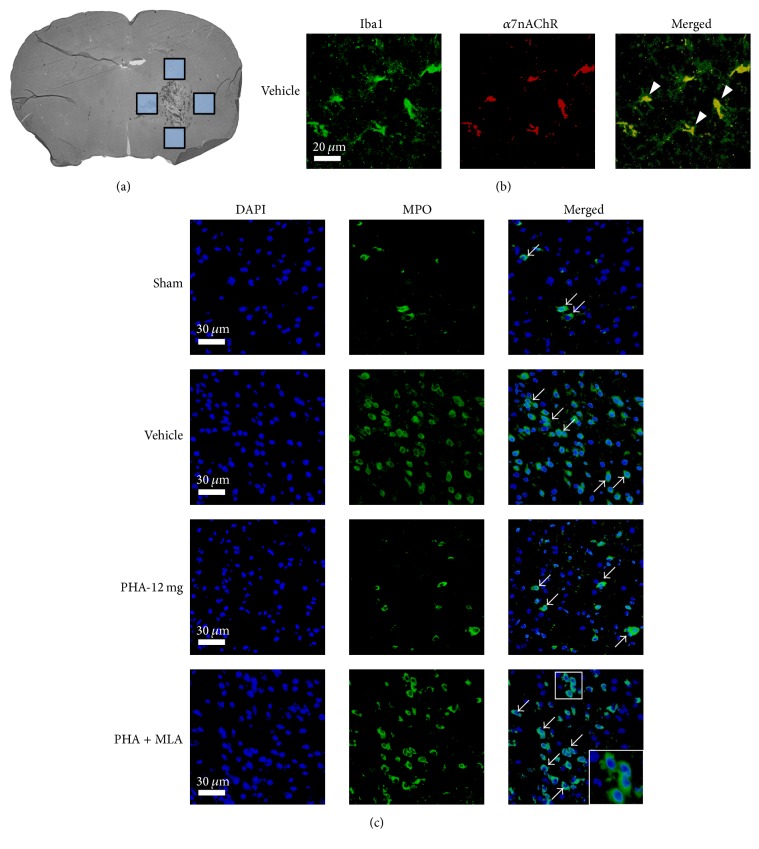
(a) Brain slice indicating the evaluated perihematomal areas (boxes). (b) Representative photomicrographs of perihematomal tissue localization of the *α*7 nicotinic acetylcholine receptor (*α*7nAChR, red) with microglia (Iba1, green). Colocalization of *α*7nAChR with Iba1 is shown in the merged image (yellow, indicated by arrowheads). Scale bar = 20 *μ*m. (c) Representative photomicrographs of perihematomal tissue with infiltrating neutrophils (MPO, green). Arrows indicate the relative changes in the number of neutrophils infiltrating for each experimental group. Scale bar = 30 *μ*m. *n* = 3/group.

Double immunofluorescence staining and imaging of perihematomal areas (Figure 2(a)) were utilized to qualitatively demonstrate localization of the *α*7nAChR on microglia (Figure 2(b)) and to demonstrate representative microphotographs of perihematomal neutrophil infiltration (Figure 2(c), *n* = 3/group). Seemingly fewer perihematomal neutrophils were seen in the PHA-12 mg group when compared to Vehicle and PHA + MLA.

### 3.2. PHA-543613 Attenuates Neurobehavioral Deficits at 24 and 72 Hours after Collagenase-Induced ICH

**Figure 3:**
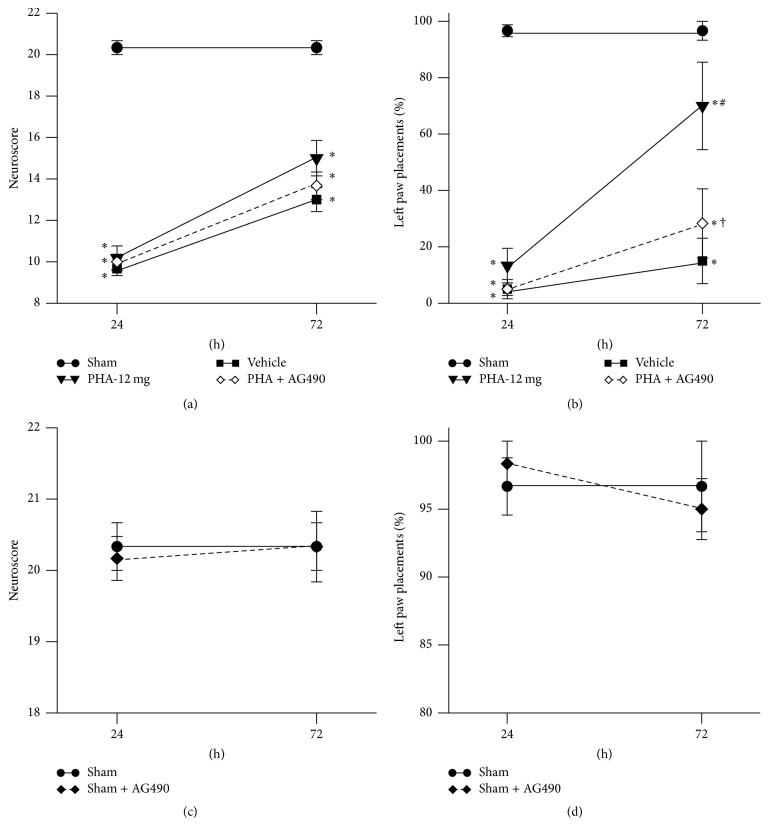
(a) Modified Garcia neuroscore 24 and 72 hours after collagenase injection. (b) Forelimb placing test 24 and 72 hours after collagenase injection. (c) Modified Garcia neuroscore for Sham controls 24 and 72 hours after surgery. (d) Forelimb placing test for Sham controls 24 and 72 hours after surgery. *n* = 6/group. Values are expressed as mean ± SEM. ^*∗*^*p* < 0.05 versus Sham, ^#^*p* < 0.05 versus Vehicle, and ^†^*p* < 0.05 versus PHA-12 mg.

At 24 and 72 hours after intrastriatal collagenase injection, neurobehavioral deficits in Sprague-Dawley rats were evaluated via the neuroscore and forelimb placement tests (Figure 3; *n* = 6/group). Rats subjected to collagenase-induced ICH demonstrated a significantly lower neuroscore than Sham animals (*p* < 0.05; Figure 3(a)); test performances between ICH animals (Vehicle, PHA-12 mg, and PHA + AG490) were indistinguishable from each other at both 24 and 72 hours after surgery (*p* > 0.05). Similarly, the forelimb placement test did not detect a significant difference in performances between ICH animals at 24 hours after ICH induction (*p* > 0.05, Figure 3(b)); however, at 72 hours after surgery PHA-543613 treated animals performed significantly better than Vehicle animals (*p* < 0.05). This treatment effect was reversed by administration of the JAK2 inhibitor, AG490 (*p* < 0.05 compared to PHA-12 mg). All rodents subjected to experimental ICH demonstrated significantly worse test performances than Sham animals (*p* < 0.05). To examine the potentially harmful effects of JAK2 antagonism, AG490 was administered to Sham animals. Neuroscore and forelimb placement test performances at 24 and 72 hours after surgery were similar between Sham animals that were given the Vehicle (DMSO diluted in PBS) and those that received AG490 (*p* > 0.05; Figures 3(c) and 3(d)).

### 3.3. PHA-543613 Attenuates Long-Term Neurobehavioral Deficits after Collagenase-Induced ICH

**Figure 4:**
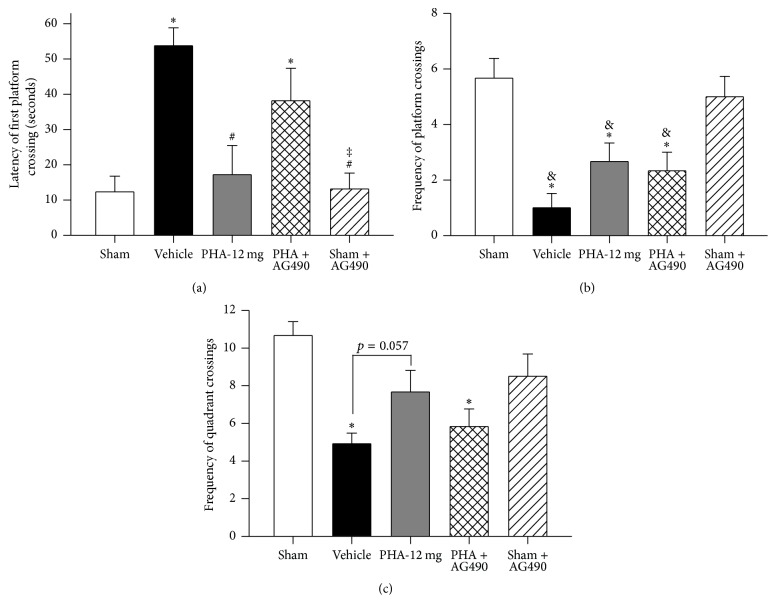
Morris water maze memory test 10 weeks after collagenase-induced ICH. (a) Latency of first platform crossing. (b) Frequency of platform crossings. (c) Frequency of quadrant crossings. *n* = 6/group. Values are expressed as mean ± SEM. ^*∗*^*p* < 0.05 versus Sham, ^#^*p* < 0.05 versus Vehicle, ^‡^*p* < 0.05 versus PHA + AG490, and ^&^*p* < 0.05 versus Sham + AG490.

Using the Morris water maze, rotarod, foot fault, and open field tests, the rat's learning ability, memory, sensorimotor function, and anxiety behavior were evaluated during the 10th week after ICH induction. The rat's learning ability and memory were tested using the Morris water maze (Figure 4; *n* = 6/group). No statistically significant difference in swim time (until reaching the platform) was detected between the experimental groups when presenting a visible or slightly submerged platform during the water maze testing (*p* > 0.05, data not shown). During the probe trials, Vehicle animals demonstrated significantly increased latencies of first platform crossing when compared to Sham (Figure 4(a); *p* < 0.05); however, PHA-543613 treatment improved the latency of first platform crossing (*p* < 0.05 compared to Vehicle, *p* > 0.05 compared to Sham). This treatment effect was reversed by AG490 (*p* < 0.05 compared to Sham, *p* > 0.05 compared to Vehicle). Sham animals demonstrated significantly more platform crossings compared to all rodents subjected to ICH (Figure 4(b); *p* < 0.05 compared to Vehicle PHA-12 mg, and PHA + AG490). Furthermore, Sham animals demonstrated significantly more quadrant crossings (quadrant of the platform) when compared to Vehicle (Figure 4(c); *p* < 0.05); however, no statistically significant differences were found between the Sham and the PHA-543613 treatment group (*p* > 0.05). AG490 reduced these treatment effects (*p* > 0.05 compared to Vehicle, *p* < 0.05 compared to PHA-12 mg). Sham animals that were given AG490 performed similarly compared to Sham animals that received administration of the Vehicle (*p* > 0.05).

**Figure 5:**
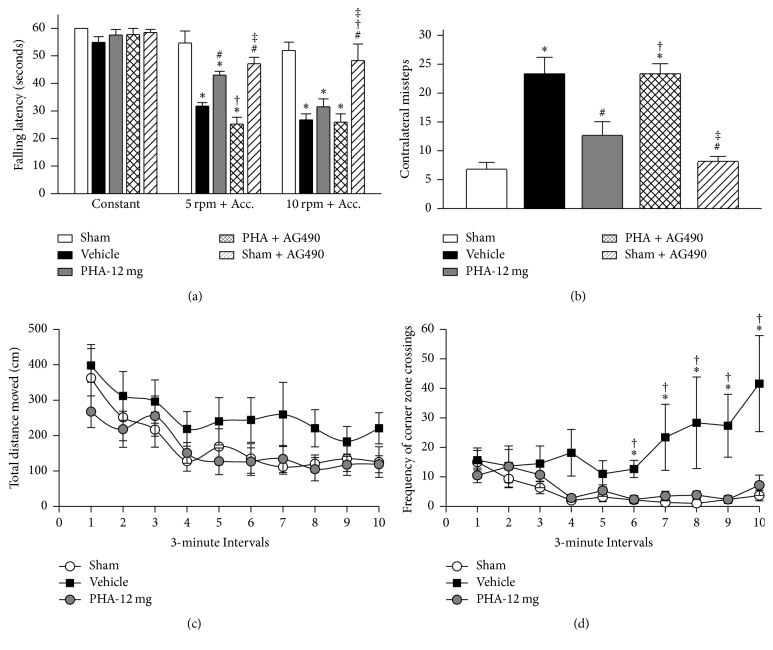
Long-term neurobehavioral tests 10 weeks after collagenase-induced ICH. (a) Rotarod falling latency. (b) Foot fault test of contralateral faults. (c) Open field distance traveled. (d) Open field frequency of corner zone crossings. *n* = 6/group. Values are expressed as mean ± SEM. ^*∗*^*p* < 0.05 versus Sham, ^#^*p* < 0.05 versus Vehicle, ^†^*p* < 0.05 versus PHA-12 mg, and ^‡^*p* < 0.05 versus PHA + AG490.

Sensorimotor function in rats was evaluated via the rotarod and foot fault tests, and anxiety behavior was evaluated using the open field test (Figure 5, *n* = 6/group). Rodents of all experimental groups demonstrated similar walking ability at a constant rotarod speed (Figure 5(a), *p* > 0.05); however, Vehicle animals demonstrated lower falling latencies compared to Sham animals during the acceleration tests (*p* < 0.05). PHA-543613 administration improved performances on the 5 rpm with acceleration test (*p* < 0.05 compared to Vehicle). The treatment effect was reversed by AG490 in the 5 rpm with acceleration test (*p* < 0.05 compared to PHA-12 mg and *p* > 0.05 compared to Vehicle). A treatment benefit was not seen at 10 rpm with acceleration as all rodents subjected to ICH performed similarly during this test (*p* > 0.05). Vehicle animals demonstrated significantly more left-sided missteps that Sham animals during foot fault testing (Figure 5(b), *p* < 0.05). The number of contralateral missteps was significantly reduced in the treatment group (*p* < 0.05 compared to Vehicle and *p* > 0.05 compared to Sham). This treatment effect was reversed by AG490 (*p* < 0.05 compared to Sham and PHA-12 mg). During the open field trials, Vehicle animals moved greater distances; however, no significant differences among any of the groups (Figure 5(c), *p* > 0.05). While experimental animals demonstrated similar numbers of corner zone crossings within the first 15 minutes of the open field test, Vehicle animals demonstrated significantly more frequent corner zone crossings than Sham and PHA-543613 treated animals within the second half of the test (Figure 5(d), *p* < 0.05).

### 3.4. PHA-543613 Reduces Brain Atrophy after Collagenase-Induced ICH

**Figure 6:**
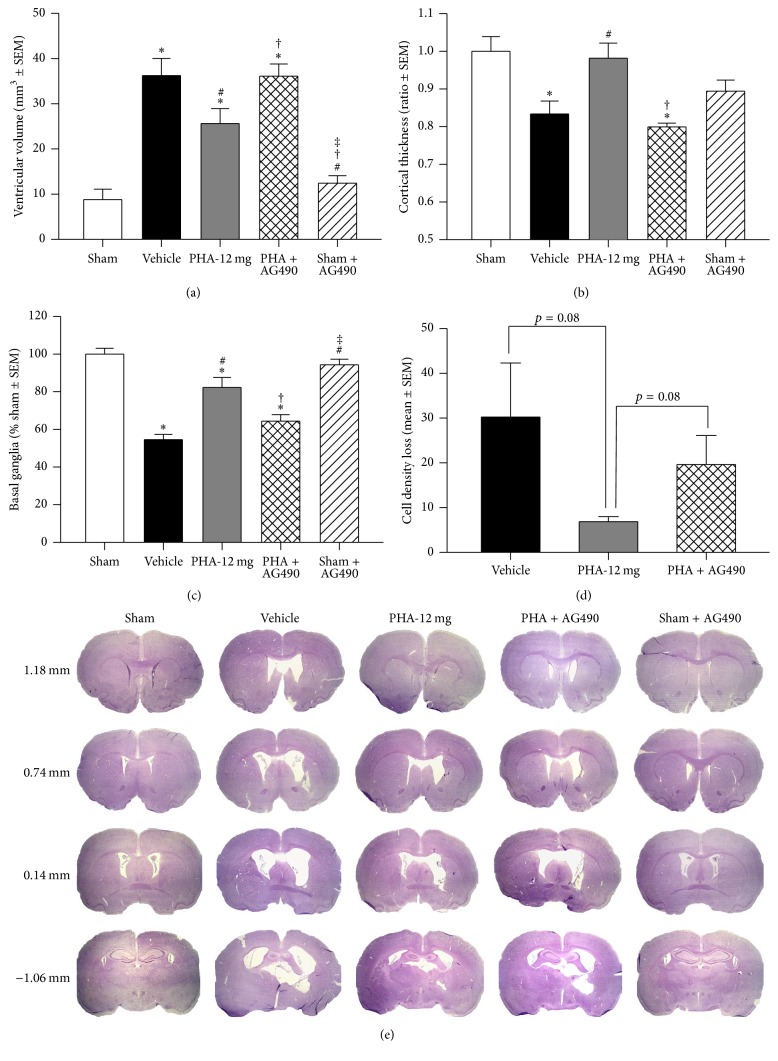
Ventricular volume (a), cortical thickness (b), basal ganglia volume (c), and cell density loss (d) after 10 weeks following collagenase-induced ICH. Representative Nissl stained brain slices (e). *n* = 6/group. ^*∗*^*p* < 0.05 versus Sham, ^#^*p* < 0.05 versus Vehicle, ^†^*p* < 0.05 versus PHA-12 mg, and ^‡^*p* < 0.05 versus PHA + AG490.

Upon completion of neurobehavioral testing at 10 weeks after surgery, all animals were sacrificed and brain tissues were collected for histologic volumetric analysis (Figure 6, *n* = 6/group). Intrastriatal injection of collagenase resulted in significantly increased ventricular volumes compared to Sham and Sham + AG490 (Figure 6(a), *p* < 0.05). PHA-543613-treated animals demonstrated significantly larger ventricular volumes when compared to Sham (*p* < 0.05); however, treated animals demonstrated smaller ventricular volumes compared to Vehicle (*p* < 0.05). Administration of AG490 reversed this treatment effect (*p* < 0.05 compared to PHA-12 mg). Similarly, cortical thickness was decreased in Vehicle animals as well as in animals that received AG450 compared to Sham and PHA-12 mg (Figure 6(b), *p* < 0.05). Volume loss was also seen within the ipsilateral basal ganglia after ICH induction (Figure 6(c), *p* < 0.05 compared to Sham). Rodents subjected to treatment with PHA-543613 demonstrated preserved basal ganglia volumes compared to Vehicle animals (*p* < 0.05), which was reversed by AG490 (*p* < 0.05 compared to PHA-12 mg). Finally, the cell density loss tended to be reduced by PHA-543613 treatment compared to Vehicle (*p* = 0.08), and this effect tended to be reversed by AG490 (Figure 6(d), *p* = 0.08 compared to PHA-12 mg and *p* > 0.05 compared to Vehicle).

## 4. Discussion

In this present study we aimed to test whether *α*7nAChR agonism, via PHA-543613, ameliorates neuroinflammation after experimental ICH through activation of the JAK2/STAT3 pathway. Indeed, administration of PHA-543613 resulted in increased protein expression of activated JAK2 and STAT3 within the ipsilateral brain hemispheres at 24 hours after ICH induction, which was associated with a decreased expression of the proinflammatory cytokine TNF-*α* and the neutrophil marker MPO. Ultimately, *α*7nAChR agonism improved neurobehavioral deficits in rodents subjected to ICH.

We conducted our experiments implementing 2 different models of ICH (intracerebral injection of autologous blood or bacterial collagenase), in 2 species (mice and rats), using 2 treatment protocols (one time versus daily administration of PHA-543613). We evaluated short- and long-term neurobehavioral function as well as histological outcomes, thereby following recommendations of the Stroke Therapy Academic Industry Roundtable (STAIR) to improve preclinical research [21]. The blood injection model, which imitates a rapidly developing intracerebral hematoma but does not involve actual vessel rupture, is often used to study perihematomal inflammation in rodents. This model does not implicate exogenous proteins (e.g., bacterial collagenase), which may aggravate cerebral inflammation. Herein, we utilized the blood injection model for our short-term experiments, establishing the *α*7nAChR/JAK2/STAT3 pathway as an anti-inflammatory treatment modality following ICH. In contrast, intracerebral injection of collagenase induces rupture of capillaries and therefore active bleeds which slowly expands over several hours similar to approximately 30% of all ICHs in patients [22]. This model is commonly used to study therapies targeting hematoma expansion. The collagenase injection model can be used to create more severe intracerebral bleeds, when compared to the blood injection model, which makes it suitable to evaluate the ability of novel therapies to reduce ICH-induced mortality or long-term neurobehavioral deficits.

Extravasated blood products promote inflammatory responses following ICH, including early activation of microglial cells and subsequent overproduction of proinflammatory mediators, resulting in recruitment and brain infiltration of peripheral leukocytes [4]. Microglial cells, specifically, play an important role in orchestrating neuroinflammation following ICH. These immune cells detect, and are activated by, bacterial pathogens and homeostatic changes associated with brain injury [23]. Activated M1 phenotype microglia produce proinflammatory cytokines such as TNF-*α*, IL-1*β*, and IL-6; M2 phenotype microglia produce brain-derived neurotrophic factor and IL-10, which has been associated with neuroprotection after brain injury [23, 24]. While microglial cells are important for the clearance of blood degradation products, the overall proinflammatory milieu attracts peripheral leukocytes to the already compromised brain region. Neutrophils, especially, were found abundantly in the perihematomal brain tissue between 1 and 7 days after ICH induction, which coincided with loss of cerebromicrovascular integrity [25]. The extent of BBB breakdown and consequent brain edema formation have been associated with poor clinical outcomes after ICH [26].

In this study we found that activated JAK2 expression was significantly higher in Vehicle than in Sham animals, which may be explained as an autogenous protective mechanism after ICH. The *α*7nAChR agonist, PHA-543613, augmented this protective mechanism. *α*7nAChRs function as ligand-gated ion channels, which promote rapid desensitizing Ca^2+^ influx [27]; yet, the mechanism of *α*7nAChR-induced JAK2/STAT3 activation is not well understood. It has been suggested that activated *α*7nAChRs have the ability to form a heterodimeric complex with JAK2, resulting in tyrosine phosphorylation and thereby activation of the nonreceptor tyrosine kinase [2, 6]. JAK2 activates STAT3, a transcription factor, which has been shown to exert its anti-inflammatory effects through IL-10. Specifically, an increase in the expression of IL-10 was inversely correlated with the expression of TNF-*α*, the latter of which has been directly implicated in neutrophil recruitment following diverse brain injury [7]. Although our study did not identify specific cell types implicated in the anti-inflammatory results of *α*7nAChR agonism, one can assume that neuronal (to a lesser extent) and nonneuronal cells (astrocytes, microglia, and endothelial cells) are implicated in this function, according to the cells' ability to produce inflammatory mediators. Furthermore, the observed anti-inflammatory effects in this study may have occurred, at least in part, through increased production of IL-10 within peripheral immune cells. Further studies are needed to determine additional underlying anti-inflammatory mechanisms of *α*7nAChR stimulation.

The competitive *α*7nAChR antagonist, *α*-bungarotoxin, has been shown to reverse *α*7nAChR-induced protection against *β*-amyloid in an in vitro model of Alzheimer's disease [28]. Since *α*-bungarotoxin does not cross the BBB [29], we used MLA, a potent and selective *α*7nAChR antagonist, to test our proposed mechanism. Indeed, MLA inhibited *α*7nAChR-mediated JAK2/STAT3 activation, which reduced or reversed the anti-inflammatory potential of PHA-543613.

*α*7nAChR agonism failed to improve neurobehavioral deficits at 24 hours after intracerebral collagenase injection, which is likely because of the severity of injury caused by this model. We implemented a 4 times higher dose of collagenase than in our previous studies [12], with the initial goal to evaluate whether PHA-543613 could reduce mortality after ICH. Surprisingly, all rodents subjected to experimental ICH survived the insult until the preselected study endpoints. Significant PHA-543613 treatment-induced improvements of neurobehavioral deficits were seen at 72 hours after collagenase injection. PHA-543613 treatment also improved long-term learning and memory abilities, as well as motor function and proprioception in rodents subjected to collagenase injection. During the water maze testing, no statistically significant difference in swim time (until reaching the platform) was detected between treated and untreated rats when the platform was visible, indicating that that results of the subsequent probe trials were likely due to changes in learning ability and memory rather than a result of motor deficits resulting in an impaired ability to swim. Furthermore, no statistically significant difference in swim time was detected between treated and untreated rats when the platform was slightly submerged, indicating that the animals found the platform either by remembering its location or by chance, before any differences could be detected. However, alterations in latency of first platform crossing and frequency of platform crossing between the experimental groups were clearly detected in the probe trials, during which removal of the platform allowed for differentiation of a focused searching behavior and random swimming within the water maze. The improved functional outcome was reflected by ameliorated histological changes within the treatment group, including decreased level of brain atrophy and a tendentially higher cell density. Similar to MLA, the selective JAK2 inhibitor, AG490, reduced or reversed the treatment effects of PHA-543613 observed at 3 days or 10 weeks after ICH. AG490 itself had no detrimental effect when given to Sham animals. These findings suggest that JAK2 plays a pivotal role in *α*7nAChR-induced neuroprotection. Interestingly, *α*7nAChR agonism affected anxiety/vigilant behavior after ICH, which we evaluated via the open field test. Sham animals demonstrated fewer corner zone crossings than animals subjected to ICH, during the second half of the open field test. This may be explained as a form of disinhibition after brain injury. In contrast, Sham and treated animals remained within the corner quadrats, which is likely the natural vigilant behavior of rodents in a new environment. Moreover, studies are needed to evaluate the neuropsychological effects of *α*7nAChR agonism after experimental ICH. Since *α*7 subunit-containing nicotinic acetylcholine receptors are also expressed within the autonomic nervous system, we have previously conducted a study evaluating the safety of systemic *α*7nAChR agonism in rats [30]. Upon administration of the *α*7nAChR agonist, PNU-282987, we measured arterial blood pressure and heart rate and collected frequent blood samples to evaluate serum pH, PO_2_, PCO_2_, Na^+^, K^+^, and Cl^−^ between 10 minutes before and 60 minutes after drug administration. We did not detect any statistically significant differences within the measurements before compared to those after drug administration.

The current study has several limitations. First, intracerebral blood and collagenase injections in rodents cannot fully reflect the ICH pathophysiology in humans. Second, we did not include dose-response experiments but utilized concentrations of drugs according to previously published protocols [12, 15]. Third, we did not include measurements of brain edema following ICH in this current study; however, our earlier experiments demonstrated that *α*7nAChR agonism decreases perihematomal brain edema formation 24 and 72 hours after ICH induction [12]. Fourth, treatments and interventions were administered systemically, which may have caused direct effects on peripheral leukocytes. Impaired activity of peripheral leukocytes following PHA-543613 administration has not been evaluated but could have contributed to the anti-inflammatory potential of this treatment. Fifth, we did not evaluate the expression of JAK2 and STAT3 and were therefore not able to demonstrate the ratios between activated/phosphorylated and total Jak2/STAT3.

Under physiological circumstances, *α*7nAChRs are involved in processes such as cell plasticity, memory, learning, locomotion, and anxiety [8]. Furthermore, alterations in receptor density or signaling were found to play an important role in neuropsychological ailments such as Alzheimer's disease, Parkinson's disease, and schizophrenia [8]. Selective stimulation of the *α*7nAChR has been shown to improve cognition in patients suffering from schizophrenia [31]. The question whether this treatment modality could benefit hemorrhagic stroke patients remains unanswered. It has been demonstrated by our work, and the work of others, that *α*7nAChR agonists exert neuroprotection in preclinical ICH models through their anti-inflammatory and antiapoptotic properties [11, 12]. Hijioka et al. showed that the *α*7nAChR agonist PNU-282987 decreased the number of activated microglial cells within the perihematomal region at 72 hours after experimental ICH induction [11].

With this current study, we showed, to our best knowledge for the first time, that the *α*7nAChR agonist PHA-543613 effectively reduced neuroinflammation via activation of the JAK2-STAT3 pathway after experimental ICH in rodents. The treatment effects of PHA-543613 included improved short- and long-term neurobehavioral deficits, which corresponded with decreased inflammatory markers at 24 hours after ICH and decreased brain atrophy at 10 weeks after ICH induction. Clinical investigations are needed to confirm the translatability of *α*7nAChR stimulation in ICH patients.

## Abbreviations

*α*7nAChR: *α*7 nicotinic acetylcholine receptor
BBB: Blood-brain barrier
ICH: Intracerebral hemorrhage
IL-6: Interleukin-6
JAK2: Janus kinase 2
PBS: Phosphate buffered saline
SEM: Standard error of the mean
STAT3: Transducer and activator of transcription 3
TNF-*α*: Tumor necrosis factor-*α*.

## Additional Points

*Highlights*. We demonstrate the role of *α*7 nicotinic acetylcholine receptor (a7nAChR) stimulation in reducing neuroinflammation after intracerebral hemorrhage by activation of the JAK2-STAT3 pathway. Our data support the notion that a7nAChR stimulation might be a novel therapeutic strategy targeting neuroinflammation after intracerebral hemorrhage, leading to a lasting functional recovery.
